# Sensory feedback can give rise to neural rotations

**DOI:** 10.7554/eLife.75469

**Published:** 2021-12-21

**Authors:** Omid G Sani, Maryam M Shanechi

**Affiliations:** 1 Ming Hsieh Department of Electrical and Computer Engineering, Viterbi School of Engineering, University of Southern California Los Angeles United States; 2 Department of Biomedical Engineering, Viterbi School of Engineering, University of Southern California Los Angeles United States; 3 Neuroscience Graduate Program, University of Southern California Los Angeles United States

**Keywords:** population dynamics, motor cortex, fronto-parietal circuits, feedback control, recurrent neural networks, Rhesus macaque

## Abstract

Investigating how an artificial network of neurons controls a simulated arm suggests that rotational patterns of activity in the motor cortex may rely on sensory feedback from the moving limb.

**Related research article** Kalidindi HT, Cross KP, Lillicrap TP, Omrani M, Falotico E, Sabes PN, Scott SH. 2021. Rotational dynamics in motor cortex are consistent with a feedback controller. *eLife* **10**:e67256. doi: 10.7554/eLife.67256

Each time you move your arm, populations of neurons in the motor cortex perform an intricate and coordinated dance that leads to the generation of movement. A large portion of this coordinated activity can be described as having a ‘rotational pattern’ over time, which is often not directly visible in the neural activity and is uncovered using dimensionality reduction methods such as principal component analysis (Figure 1A; Churchland et al., 2012). Such rotational dynamics have been observed in the motor cortex in many studies involving arm reaching or reach and grasp movements (see, for example Kao et al., 2015; Pandarinath et al., 2015; Suresh et al., 2020; Susilaradeya et al., 2019; Abbaspourazad et al., 2021; Sani et al., 2021), but were absent in the supplementary motor area (Lara et al., 2018).

**Figure 1.**
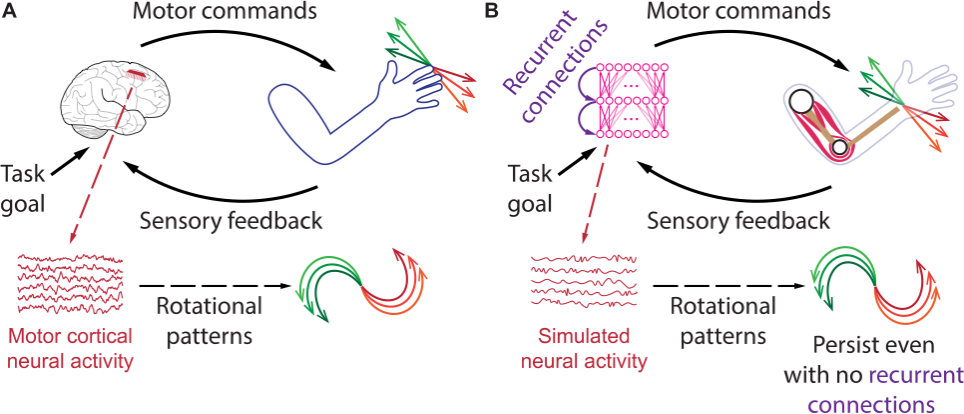
Using an artificial network to investigate how rotational patterns are generated in the motor cortex. (**A**) The brain and the arm together can be viewed as a closed-loop feedback control system. When the brain receives instructions for a task, neurons in the motor cortex (red inset) send a command to the arm, which moves and returns sensory information back to the cortex. During arm movements, the activity of neurons in the motor cortex exhibits rotational patterns, which may not be visible directly, but usually emerge after neural activity (red graph) has been subjected to dimensionality reduction methods and averaged across several repetitions of the same movement (different movements are shown with different colors). (**B**) A similar closed-loop system can be constructed in simulations with an artificial neural network (magenta, left) replacing the brain and a musculoskeletal model (right) replacing the arm. Kalidindi et al. show that such a system generates rotational patterns in the artificial neural network that resemble those observed in the motor cortex, regardless of the presence or absence of recurrent connections (purple).

Several studies have investigated the mathematics behind how these rotational patterns arise from the activity of individual neurons (e.g., Michaels et al., 2016; Elsayed and Cunningham, 2017). Some of these reports (Sussillo et al., 2015; Michaels et al., 2016) rely on artificial neural networks: simplified computational representations of interconnected neurons, or groups of neurons, that allow researchers to study how patterns of activity in the brain may emerge. These networks make it possible to explore how the nervous system might perform certain tasks without using real brains, which are harder to observe and difficult or impractical to manipulate.

One question that remains a topic of lively investigation is whether rotational patterns of activity are generated autonomously within the motor cortex itself or whether they reflect ongoing inputs from other regions of the brain (Vyas et al., 2020). It is known that networks with recurrent connections – this is, networks with ‘memory’, in which the output can be affected by previous inputs – can generate patterns autonomously. Indeed, a group of researchers discovered that when they trained a recurrent artificial network to generate the motor activities needed for movement, the patterns resembled the rotational activity seen in the motor cortex (Sussillo et al., 2015). Now, in eLife, Hari Teja Kalidindi (Scuola Superiore Sant'Anna), Kevin P Cross (Queen's University) and colleagues report that it is also possible to train a neural network to control an artificial arm without using any recurrent connections inside the network (Kalidindi et al., 2021).

The team (who are based in Italy, Canada, the United Kingdom and the United States) constructed an artificial neural network that can activate muscles on a simulated arm which then sends sensory information, such as its position and muscle activations, back into the network (Figure 1B). The artificial neural network was trained to perform reaching movements or to counter disturbances from the environment, such as forces that suddenly pushed the arm aside. This approach replicated key findings from non-human primate experiments in that the activity of neurons in the brain showed rotational patterns, whereas muscle activations in the arm did not. Intriguingly, Kalidindi et al. also found that when they stripped recurrent connections from the neural network, it could still learn to move the artificial arm, and still generated rotational patterns in its activity.

So how can a network with absolutely no recurrent connections produce rotational patterns similar to those observed in the motor cortex? Since non-recurrent networks always return the same output when they receive a specific input, the only way they can produce patterns that vary over time is if the input to the network also changes over time. Indeed, Kalidindi et al. found that the sensory feedback signals from the arm, which act as the input to the neural network, also show rotational patterns. Thus, feedback from the arm’s position and from muscle activations is sufficient to generate rotational patterns of activity in the brain. When Kalidindi et al. repeated the experiment with monkeys performing the same tasks, they observed rotational patterns not only in the motor cortex, but also in the somatosensory cortex, the region of the brain that receives and processes sensory information from the environment. This suggests that sensory feedback to the real brain may also contain rotational dynamics, as was the case in the artificial network simulations.

Another important consideration is the structure of the behavioral task. Usually, the tasks used to study the activity of the motor cortex involve the arm being stationary at an initial position and ending up stationary in another. So, could the rotational patterns observed in these tasks be due to the movement starting and ending at the same zero-velocity state? To investigate, Kalidindi et al. trained neural networks to perform a new task in which the arm continuously tracks a target moving at a constant velocity. Both recurrent and non-recurrent networks performed well, but this experiment led to substantially less rotational dynamics than the previous tasks, suggesting that the design of the behavioral task can play a critical role in the prominence of rotational patterns in neural activity.

The work of Kalidindi et al. cleverly uses artificial neural networks and real-world data to highlight the importance of studying the motor cortex in the context of the entire closed feedback loop between the brain and the body, and the need to study this system using different types of tasks. Critically, the experiments suggest that rotational patterns observed in the motor cortex can be influenced not only by internal autonomous dynamics, but also by external inputs such as sensory feedback. An important future research direction is to tease apart the extent to which these two contributing factors influence neural activity in the motor cortex.
